# Development of a Casting Process Database for Rapid Process Design Using Case-Based Reasoning

**DOI:** 10.3390/ma18030505

**Published:** 2025-01-23

**Authors:** Chuhao Zhou, Shuren Guo, Dong Xiang, Huatang Cao, Beibei Li, Yansong Ding, Xuanpu Dong

**Affiliations:** State Key Laboratory of Materials Processing and Die & Mould Technology, School of Materials Science and Engineering, Huazhong University of Science and Technology, Wuhan 430074, China; zhouch09@hust.edu.cn (C.Z.); caoht@hust.edu.cn (H.C.);

**Keywords:** casting process design, database, intelligent manufacturing, case-based reasoning, shape feature extraction, gating system

## Abstract

Casting process design is crucial in manufacturing; however, traditional design workflows are time-consuming and seriously reliant on the experience and expertise of designers. To overcome these challenges, database technology has emerged as a promising solution to optimize the design process and enhance efficiency. However, conventional database storing process cases often lack adequate parametric information, limiting their ability to support intelligent and automated design. Thus, this study has developed a casting process database based on parametric case modeling, enabling the rapid design of casting processes for new parts using case-based reasoning (CBR). The database framework was designed to organize process cases into four distinct information modules, allowing for structured and separated storage. Data unit associations were established between these modules to ensure the completeness and scalability of process information. The database stores sufficient parametric information to describe key process characteristics and multidimensional elements. This includes the detailed structural parameters of parts, calculated based on the accurate analysis of their structural features. A process cost estimation model was incorporated to calculate and record direct process costs, enabling the effective comparison and ranking of various process plans for the same part. Additionally, the parametric model of the gating system is stored to support the transfer of processes between similar parts. The functionality and effectiveness of the proposed database were visually validated through a case study on the process design of an actual casting part. The results indicate that the database significantly improves efficiency and ensures the accuracy of CBR-based process design while optimizing the reuse of design knowledge and expertise. The developed database achieved a 90% reduction in design time compared to conventional methods.

## 1. Introduction

The design of casting processes is a complex and demanding task, encompassing multiple aspects such as gating system design, riser design, and cooling process control. Due to the numerous factors involved in casting, such as material properties, casting geometry, and production conditions, the design process requires a combination of theoretical knowledge and extensive practical experience. For instance, the dimensions of the gating system must be determined using appropriate formulas based on the physical characteristics of different materials (e.g., melting point, fluidity, and shrinkage rate), while also referencing empirical tables and adjusting correction factors according to operating conditions. These calculations, along with the subsequent verification of results, make the entire process not only highly intricate but also mechanical in nature. Similarly, casting riser design is significantly influenced by the solidification and shrinkage characteristics of the material as well as the geometric structure of the casting. The size and placement of risers are directly related to the quality of the final casting, and this process heavily relies on the expertise and practical experience of designers. Although foundry enterprises are increasingly adopting computer simulation technologies to optimize process design, the mechanical nature of table lookups, complex calculations, and the indispensable role of designers’ experience continue to play a critical role in casting processes. Consequently, integrating digital technologies into casting process design is essential for improving design efficiency and reducing reliance on manual labor.

Currently, most casting process databases, developed for the accelerated design and intelligent optimization of casting parameters, are built within conventional process design workflows. These databases primarily store design parameters and rules pertinent to conventional process design workflows, while some studies integrate optimization algorithms to further improve the processes. Song et al. [1], Wang et al. [2], and Wang et al. [3] established a casting process database utilizing a Structured Query Language (SQL) Server, which systematically arranges relevant process parameters in a tabular format to facilitate rapid queries and computations. Liu et al. [4] and Xiang et al. [5,6] developed a casting process database that integrates Computer Aid Design (CAD) technology, enabling the rapid design of processes and model creation. Deng et al. [7] developed a database for die-casting process parameters, optimizing these parameters using the principle of similarity and an enhanced K-Nearest Neighbor (KNN) algorithm. Suthar et al. [8] established a database for parameters in sand casting and investment casting and executed parameter optimization through clustering algorithms. Chen et al. [9] cataloged the relationship between casting techniques and geometric forms in a database to facilitate subsequent process design. Claudio et al. [10] archived empirical and tacit knowledge from casting process design and practical production in a database and combined these data with 3D model geometric feature-recognition technology to improve the processes. Zeng et al. [11] utilized text information extraction techniques to obtain data pertinent to squeeze casting processes from journal literature, subsequently storing this information as rules in a database to drive process design. These casting process databases developed for the rapid design and intelligent optimization of casting process parameters are primarily used to store and manage process design parameters, design rules, and knowledge related to actual production, while leveraging digital methods to enable the rapid determination and optimization of the design process. However, the design logic of such databases essentially inherits the framework of traditional process design workflows, whereby process parameters are deduced step by step in a fixed sequence. For instance, casting process parameters are determined based on production conditions and part dimensions, followed by the determination of gating system dimensions based on material properties, then the dimensions and placement of risers are established according to the part geometry, and, finally, results are validated and adjusted in conjunction with simulation or actual production results. Although these databases enhance efficiency through automated calculations and rule matching, they have not overcome the inherent complexity of traditional design workflows. The design process still requires layer-by-layer calculations following established rules (e.g., using empirical formulas, parameter tables, and correction factors), with results sequentially derived according to the traditional design order. As a result, these databases fail to fundamentally simplify the design process to improve efficiency. Moreover, the updating and iteration of such databases rely on the continuous accumulation of theoretical knowledge and expert experience, which serve as the foundation for defining rules. However, breakthroughs in theoretical and empirical principles in the field of casting are difficult to achieve in a short period, thus imposing certain limitations on the evolution of these databases. Consequently, a more effective approach is required to enhance process design efficiency.

Case-based reasoning (CBR) is a methodology that offers solutions to current problems derived from historical case experiences [12,13,14,15]. The fundamental concept is to search the case database for the historical case most similar to the cases at hand, leveraging the experiences and strategies from that case guide problem resolution. In casting production, numerous successful practical cases exist. Consequently, during the development of a casting process for a new part, the most analogous part can be identified in the case database to enable an accelerated design process. This approach minimizes design steps and increases design efficiency by reusing established process solutions and fully utilizing prior experience.

In traditional databases which archive casting process cases, key elements within cases—such as the geometric features of parts or the design characteristics of process models—are typically described and stored in the form of keywords or text fields [16]. While this approach is intuitive, it has several drawbacks, including a lack of unified constraint rules, high flexibility in creation and modification, poor data stability, vague semantic representation, and difficulty in achieving structured representation. On the one hand, manually defined keyword descriptions often fail to accurately and comprehensively reflect the geometric features of parts, resulting in low retrieval efficiency and accuracy for similar parts. Studies have shown that frequent interactions during the retrieval process led to design time consumption accounting for more than 60% of the total design time [17]. On the other hand, non-parametric feature descriptions limit the efficiency and accuracy of process model reuse. For example, during the reuse of gating systems, the process typically relies on manually entered dimensional data at the time of database entry or referencing stored 3D models to construct the gating system for new parts. However, manually entered data are often incomplete, making it difficult to accurately describe the geometry and positional information of the entire gating system model. Similarly, stored 3D models are challenging to extract efficiently and often lack specific parametric information. Furthermore, during reuse, adjustments to the dimensions of the gating system from the source part are necessary to accommodate the requirements of the new part. However, manually entered data or stored 3D models typically cannot be quickly modified using specific correction algorithms, significantly impacting the efficiency and accuracy of the reuse process.

This study performs a comprehensive analysis of the parametric representation of part and process information and subsequently develops a casting process database intended for rapid CBR-based process design. The database retains parametric information that accurately represents the shape–structure features of the parts through precise calculations. A process cost estimation model is developed to compute and store the direct costs associated with the process, enabling the ranking of various process plans for the same part. The database stores parametric models of the gating system, supporting the efficient transfer of mature processes to new parts. Meanwhile, the storage structure of the database is designed and optimized to associate various types of information involved in process design, allowing for the comprehensive storage and description of entire process cases, thereby minimizing information redundancy and promoting the sharing and reuse of process information.

The study is organized as follows. Section 2 introduces the storage structure and the core content of the database. Section 3 details the parameterization of process information, with a focus on three key aspects: the structural feature extraction of part shapes, process cost calculation, and the parametric modeling of the gating system. Section 4 illustrates the application of the database and validates its feasibility for the casting process design of new parts, followed by detailed results analysis. Finally, Section 5 concludes the study by summarizing the findings and presenting prospects for future work.

## 2. Database Architecture and Data Storage Design

### 2.1. Database Development Technology Framework

The database system was implemented using the Visual Studio 2022 development environment, C# programming language, and MongoDB (version 6.0) as the database management system.

At present, descriptor-based 3D model retrieval technology is developing rapidly. The core of this technology lies in constructing feature descriptors to extract the key features of 3D models. Feature descriptors uniquely identify models through feature extraction and rank them during retrieval by calculating the feature space distance between different parts, treating models with closer feature distances as similar models. To support this technology, databases need to efficiently store these feature descriptors. Specifically, for castings with cavities and complex geometric structures, the data structure of the descriptors is often quite intricate. Moreover, each part may correspond to multiple casting process schemes, so the database should also have good scalability to accommodate the dynamic expansion of process data units.

MongoDB, a document-oriented NoSQL database, contrasts with conventional relational databases like SQL, which store structured data in fixed schemas organized as tables. Instead, MongoDB employs BSON (Binary JSON) to store data, enabling support for diverse data types and complex hierarchical structures. Its flexible schema design allows dynamic data model adjustments, significantly enhancing storage flexibility.

In this system, MongoDB was adopted as the primary data storage solution due to its high scalability and ability to model complex data. This enables the efficient storage and retrieval of parts’ shape–structure features and process parameters. The database architecture adopted a modular and hierarchical design, favorably facilitating efficient management and seamless expansion of part information along with associated process data.

### 2.2. Database Functionality Analysis

**Figure 1:**
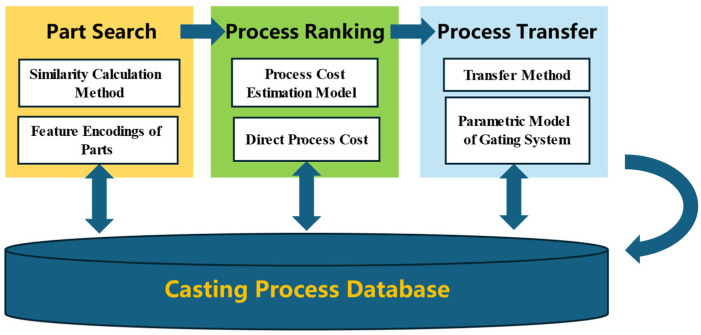
Functionality of the casting process database.

For the accelerated design of casting processes using CBR, the casting process database must contain three operational modules: part retrieval, process cost estimation, and process transfer, as illustrated in Figure 1. In the part retrieval module, it is essential to retrieve several parts that closely resemble the structure and shape of the new part. Therefore, the database must store information regarding the shape and structural features of the parts to support similarity assessments between different parts. The retrieved similar parts may have multiple processes stored in the database, such as using different casting methods or adopting different gating system layouts. Thus, these different process plans should be ranked to quickly find the best process corresponding to that part. To rank process plans from a cost perspective, a process cost estimation model can be employed to calculate an approximate cost for each plan as it is entered into the database, with the cost stored alongside the process plan. Upon identifying the optimal process plan for a similar part, it should be straightforward and efficient to utilize the plan as a reference for designing the process plan for the new part. Considering that the structure and dimensions of the gating system are important components of the process plan, the process database should store a parametric model of the gating system for each process. Thus, during process migration, the parameter model of the source part can be directly modified and optimized as required, and subsequently expeditiously applied to a similar new part.

### 2.3. Database Storage Structure Design

**Figure 2:**
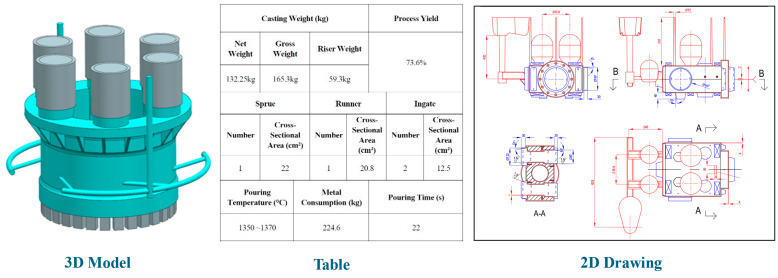
Schematic of data components in a complete process case.

A complete process case contains various types of information, such as 3D models, 2D drawings, process parameter tables, operation workflows, quality inspection reports, and historical production data, as illustrated in Figure 2. Currently, when storing this complex and diverse information, a tree-like directory structure is generally adopted. However, this traditional storage structure has obvious limitations. For instance, when a part or standardized riser component is associated with multiple casting processes, this information may be repeatedly stored, resulting in information redundancy, wasted storage space, and heightened management complexity. In addition, the hierarchical directory structure is inefficient for part retrieval. Users must navigate the intricate directory hierarchy sequentially to locate specific process information, thereby augmenting the complexity and time expenditure of retrieval. This study adopts MongoDB as the foundational solution to address the aforementioned issues, leveraging its flexible document model and robust data processing capabilities to design a novel storage structure for complete process cases. This approach reduces information redundancy, simplifies retrieval, and improves data management efficiency.

**Figure 3:**
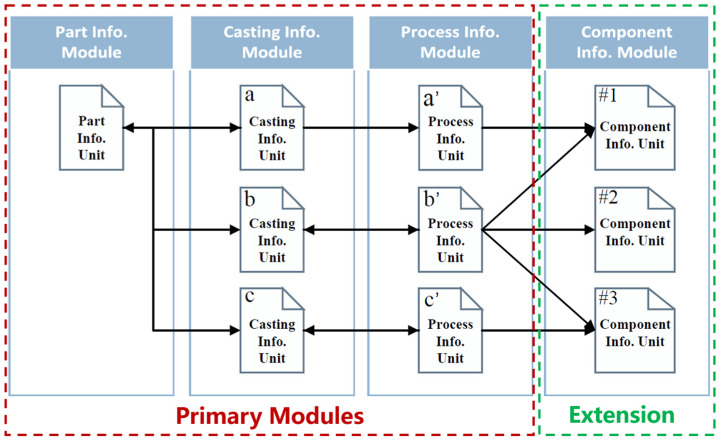
Relationships between data units across different modules.

In this study, the database is divided into four modules: the part information module, casting information module, process information module, and standard component information module. Each part, casting, and process entered into the database was processed as a Bson formatted document and stored separately in the three main modules, forming the basic units of each information module. If the components involved in the process, such as risers and chills, were standard, they were marked and linked to the corresponding data units in the standard component information module. Figure 3 illustrates the relationships between data units across various modules.

Due to variations in casting methods and process plans, a single part can be associated with multiple processes. As a result, the part information units and process information units in the database exhibit a one-to-many relationship. On the other hand, each process plan corresponds to a specific casting with precisely determined process parameters. Therefore, the casting information units and process information units form a one-to-one relationship. Additionally, each process plan may refer to multiple standard components, such as risers and chills, resulting in a many-to-many relationship between the process information units and the standard component information units.

**Figure 4:**
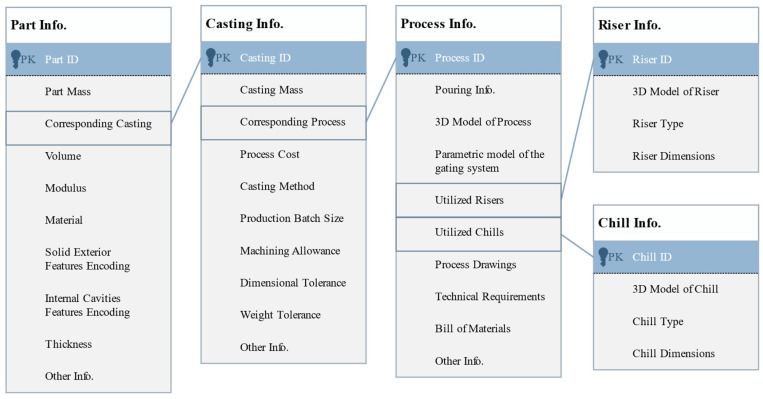
Stored content of a process case in the database.

The main contents of the process case stored in the database are shown in Figure 4. By linking the corresponding data units from the four information modules—parts, castings, processes, and standard components—a complete process case is formed. Additionally, distinguishing between part information, casting information, and process information enables the effective management and storage of relevant data. This approach helps prevent data loss, reduces redundancy, and makes information classification more explicit, thereby improving the efficiency and accuracy of process information management.

The part information module mainly included information such as part ID, material, volume, modulus, encodings, etc. Among them, the key information was volume, modulus, solid exterior features encoding and internal cavities features encoding, based on which the similarity of the shape–structure features between different parts could be calculated.

The casting information module mainly included information such as casting ID, mass, material, casting method, production batch size, machining allowance, dimensional tolerance, weight tolerance, direct process cost of corresponding process, etc.

The process information module primarily encompassed details such as process ID, 3D process model, 2D drawings, process instructions, bill of materials, parametric model of the gating system, utilized standard components, etc. Among them, the parametric gating system model simplifies the reuse of process plans.

The standard component information module for sand casting primarily comprises two sub-modules: riser information and chill information, which contain detailed design parameters for risers and chills, respectively. During the storage procedure, the process information unit established an association with the corresponding data units in the standard component module, ensuring the data integrity of the overall process plan. At the same time, this storage structure enhanced the scalability and maintainability of standard components in the process plan within the database.

## 3. Parametric Representation and Storage of Process Information for Rapid Design

The database must ensure data integrity, meaning that the stored process information should encompass a complete process case to guarantee comprehensive data entry and the convenient retrieval of process plans. Furthermore, the saved data must include sufficient parametric information to thoroughly delineate essential process characteristics and multidimensional aspects. Implementing parametric representation can substantially diminish the necessity for manual definitions, hence improving the digitalization of process information management. This approach increases the database’s capability, offering critical data support for the rapid design of new processes through the reutilization of previous ones.

### 3.1. Extraction and Storage of Part Shape–Structure Features

**Figure 5:**
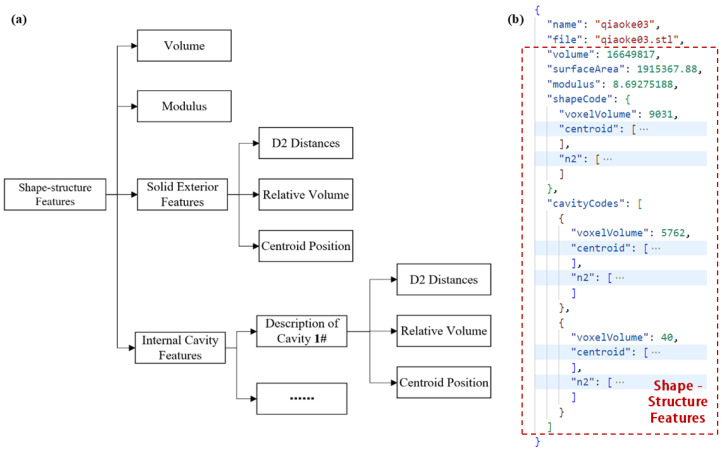
(**a**) Structure of part-features encoding; (**b**) detailed content of features encoding.

The key step in achieving fast design via CBR is the precise retrieval of casting parts from the database. To achieve this, it is necessary to store data that can describe the structural and shape features of the parts to support the similarity calculation between the two parts. This mainly includes the following three aspects:

1. Storing the volume of the part. The quality grade and volume of the part influence various factors, including the positioning and dimensions of the gating system, as well as the volume and configuration of the risers. When the volume difference exceeds a certain range, notable variations will occur in the applicable process plans for different castings.
2. Storing the average modulus of the part. The average modulus is the ratio of the part’s volume to its surface area, which is an important parameter characterizing its heat-dissipation ability during the solidification process. Since the average modulus influences the cross-sectional thickness and heat-dissipation rate of the part, there are significant differences in the distribution of hot spots and temperature fields during the solidification process for parts with large variations in modulus. The aforementioned changes will substantially impact the design of the process plan, particularly the layout of the gating system and risers.
3. Storing the feature information of the solid exterior and internal cavities of the part. Since the size, shape, and relative position of the mold cavity and cores during the casting process determine the shape and structure of the formed part, the shape features of the part can be represented by a combination of the outer shape features representing the mold cavity and several inner cavity features representing the cores. A combination of relative volume, centroid position, and the D2 distance descriptor proposed by Osada et al. [18] were used to encode the outer shape and inner cavity features, respectively. The calculated outer shape features describe the overall dimensions, contours, and other macroscopic characteristics of the part, while the inner cavity features reflect the internal complex structure, wall thickness distribution, and other microscopic details of the part, to a certain extent. Figure 5 shows the structure and content of the features’ encoding.

**Figure 6:**
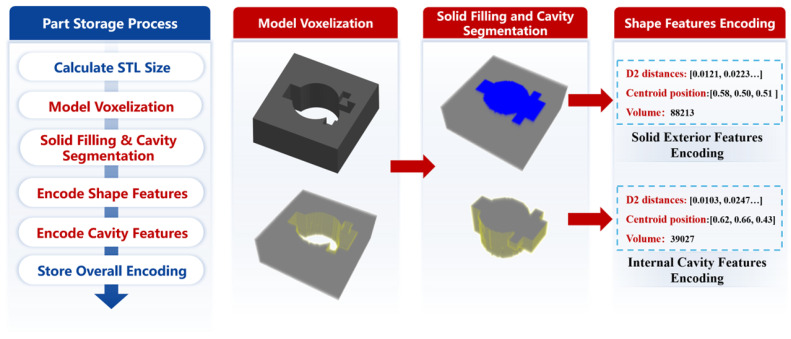
Workflow for part storage and encoding.

The procedure for storing parts is illustrated in Figure 6. Initially, the target part is imported, and the dimensions of the Standard Tessellation Language (STL) model are calculated, primarily determining the volume and average modulus of the model. Subsequently, the model data undergoes preprocessing. Considering that parts in casting production usually have complex cavity structures, the voxelization method is chosen to describe the geometric shape of the model and depict the complex internal structures in the 3D model. Secondly, based on the voxel model of the part, the algorithm in reference [19] is used to extract the solid outer shape and internal cavities of the part. Following that, the features of the solid exterior and internal cavities are encoded. Ultimately, by integrating data such as volume, modulus, solid exterior features, and internal cavity features, a comprehensive feature encoding that describes the structural and shape information of the part is generated and preserved within the corresponding data unit of the part information module. Using this encoding, similarity assessments can be conducted for different parts [19].

### 3.2. Construction of Process Cost Estimation Model

By calculating the similarity of parts, the most similar part to the new part to be designed can be retrieved from the database. According to the storage structure of the database, this part may correspond to multiple casting process plans. Therefore, it is essential to rank these different process plans to determine the optimal one. This study aims to rank mature processes, which produce parts devoid of significant casting defects and meet technical requirements, based on economic considerations. Taking sand casting as an example, a process cost estimation model is constructed to implement this approach.

In general, cost estimation in the casting production process involves multiple factors, including material costs, labor expenditures, energy costs, mold expenses, plant costs, equipment depreciation, etc. [20,21,22,23]. The direct costs of process design primarily include material expenses (mainly determined by weight) and processing costs (evaluated based on energy consumption) for the estimation and comparison of various process plans.

The direct process cost model constructed in this study exclusively accounts for material and energy consumption, both of which are directly driven by the process plans and design parameters. To accurately quantify the casting process cost, this study has developed a calculation method based on these factors. The calculation method for the casting process cost $C_{\mathrm{castingProcess}}$ is as follows:

(1) $$C_{\mathrm{castingProcess}}=\left(C_{\mathrm{material}}+C_{\mathrm{energy}}\right){/n}_{c}$$

where $C_{\mathrm{material}}$ is the material cost, $C_{\mathrm{energy}}$ is the energy cost, and $n_{c}$ denotes the number of castings included in the process (i.e., the number of cavities in the mold). It is essential to note that the estimation of process costs in this study excludes equipment status and actual production conditions; thus, the calculation model does not account for material and energy losses resulting from scrap at each stage of casting production, nor does it consider the effects of scrap recycling on costs.

The material cost comprises two components: direct materials and indirect materials. Direct materials refer to the components that constitute the final cast metal product, including the casting, gating system, and risers. Indirect materials are utilized to form the casting and enhance casting quality during the process, including sand, filters, riser sleeves, chills, and various auxiliary materials. The formula for determining material cost is as follows:

(2) $$C_{\mathrm{material}}{=C}_{\mathrm{direct}}+C_{\mathrm{indirect}}$$

The formula for calculating direct material cost $C_{\mathrm{direct}}$ is:

(3) $$C_{\mathrm{direct}}{=c}_{\mathrm{unit}\text{\_}\mathrm{metal}}\;\times \;\left(w_{\mathrm{cast}}+w_{\mathrm{gatingSystem}}+w_{\mathrm{riser}}\right)$$

where $c_{\mathrm{unit}\text{\_}\mathrm{metal}}$ is the cost per unit weight of metal, $w_{\mathrm{cast}}$ is the weight of the casting, $w_{\mathrm{gatingSystem}}$ is the weight of the gating system, and $w_{\mathrm{riser}}$ is the total weight of all risers in the process.

The formula for calculating indirect material cost $C_{\mathrm{indirect}}$ is:

(4) $$C_{\mathrm{indirect}}{=c}_{\mathrm{unit}\text{\_}\mathrm{sand}}{\;\times \;\rho }_{\mathrm{sand}}\;\times \;\sum_{i=1}^{3}\left(x_{i}+t_{\mathrm{mold}}\right)+c_{\mathrm{filter}}+c_{\mathrm{sleeve}}+c_{\mathrm{chill}}$$

where $c_{\mathrm{unit}\text{\_}\mathrm{sand}}$ is the cost per unit mass of sand (including sand, binders, and coatings), $\rho_{\mathrm{sand}}$ is the density of the sand, $x_{i}$ represents the dimensions of the process model’s bounding box in the i-th direction, $t_{\mathrm{mold}}$ is the average mold thickness, $c_{\mathrm{filter}}$ is the cost of the filter, $c_{\mathrm{sleeve}}$ is the cost of the riser sleeve, and $c_{\mathrm{chill}}$ is the total cost of all chills used in the process.

Energy consumption during the casting process is concentrated in several stages: metal melting, molding, core fabrication, pouring, and subsequent cleaning and machining. The melting process is significant in the overall energy consumption of the casting, necessitating substantial electricity or fuel to heat the metal to its melting point and sustain it in a liquid form. This study simplifies the calculation methodology by focusing just on the energy consumption of the melting process for estimating direct process costs, excluding equipment considerations such as energy conversion efficiency. The energy necessary to melt the metal can be approximated using a thermodynamic equation:

(5) $$C_{\mathrm{energy}}=c_{\mathrm{unit}\text{\_}\mathrm{energy}}{\;\times \;w}_{\mathrm{metal}}\;\times \;\left[c_{\mathrm{ps}}\;\times \;\left(t_{\mathrm{melt}}{\;-\;t}_{\mathrm{room}}\right)+{L+c}_{\mathrm{pl}}\;\times \;\left(t_{\mathrm{tap}}-t_{\mathrm{melt}}\right)\right]$$

where $c_{\mathrm{unit}\text{\_}\mathrm{energy}}$ represents the cost per unit of energy, $w_{\mathrm{metal}}$ is the mass of metal consumed in the process, $c_{\mathrm{ps}}$ and $c_{\mathrm{pl}}$ are the specific heat capacities of the metal in its solid and liquid states, respectively, L is the latent heat of fusion, $t_{\mathrm{room}}$ is the room temperature, $t_{\mathrm{melt}}$ is the melting temperature of the metal, and $t_{\mathrm{tap}}$ is the tapping temperature.

Data related to the process model, including model dimensions, the mass of the casting and gating systems, and filter and chill information, can be retrieved from the process database. Based on these data, the direct cost of the process can be computed and recorded as a label within the process data unit. This will provide essential data support for the comparison and selection of different process plans in subsequent stages.

### 3.3. Storage of Parametric Models for Gating Systems

As the channel through which molten metal is introduced into the mold cavity, the gating system plays a critical role in process design. Thus, establishing the parameterization of the gating system is beneficial to boosting the intelligence of process design and the digitization of the database. The gating system can be conceptualized as a network of interconnected pipes that constitutes a directed acyclic graph (DAG) structure [24]. This structure can be described as G(V,E), where V represents the collection of nodes and E denotes the set of directed edges. In this representation, the nodes correspond to the basic pipeline segments that make up the gating system model. These segments are sequentially connected to ultimately form the sprue, runner, and ingate. The edges represent the connections between different pipeline segments. In a pair of nodes, *u* is referred to as the tail, *v* is the head, and the edge *e* is termed the outgoing arc from *u* and the incoming arc to *v*. Incorporating casting knowledge, the molten metal flows into the gating system via the sprue and exits through the ingate. Therefore, the node with no incoming arcs represents the sprue, while the node with no outgoing arcs represents the ingate. This structured and parametric gating system is more conducive to analysis and computation in a computer context than images or models.

**Figure 7:**
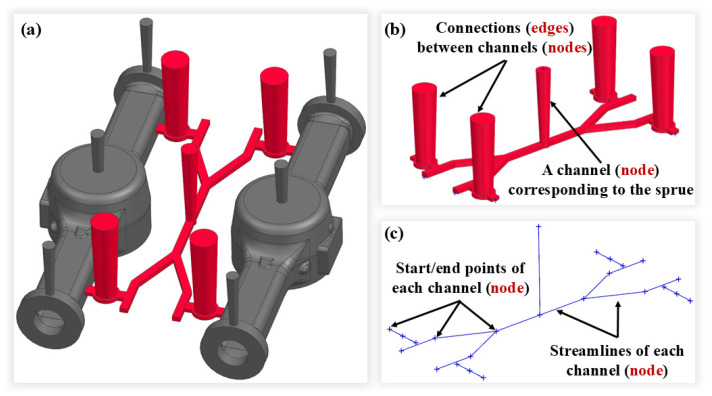
(**a**) The process for an axle housing; (**b**) the 3D model and graph structure of the gating system; (**c**) the model created by the streamlines.

Figure 7a shows the process for an axle housing part, with the gating system colored in red. From the perspective of traditional 3D modeling, the gating system is viewed as a series of pipe segments, each of which is formed by sweeping its cross-section along a streamline (either straight or curved). In Figure 7b, the sprue of the gating system is seen as a singular pipe, while the runner has several pipe segments. Each pipe segment is denoted by a node in the DAG, while the interconnections among the pipe segments are illustrated by directed edges between the nodes. The linkage between various pipe segments is generally either a natural connection or a lap joint. For these common connections, the corresponding edges do not require supplementary information; the local connection characteristics can be fully expressed using the data of adjacent nodes. For special connections, such as the open riser located at the intersection of two pipe segments on the ingate, it is essential to document the riser’s feature information on the corresponding edge. Figure 7c displays the model created by the streamlines of the different pipe segments within the gating system. The streamline is determined by two points (for an arc streamline, the center and radius are used), making the positional data of these points a critical part of the node data, i.e., the pipeline segment’s information.

In practical software implementations, DAGs are typically stored using data structures such as adjacency lists or adjacency matrices. In the adjacency list model, each node in the graph corresponds to a linked list that stores the target nodes of all edges directly connected to that node. Adjacency lists are efficient for representing sparse graphs, as they save storage space and facilitate dynamic operations on nodes and edges. For the gating system, given its relatively small scale, limited number of nodes and edges, and primarily tree-like topology, the graph’s structure and depth are not complex. To facilitate the addition or deletion of nodes and to allow for the extension of node-related attributes, an adjacency list is a more suitable data structure for storage.

Leveraging the characteristics of the gating system and the flexible document storage capabilities of MongoDB, this study proposes several improvements to the traditional adjacency list model:

- Storing references to both parent and child nodes in each node: When constructing a new gating system model by referencing the DAG data of a source part, an effective and reliable method is to perform a reverse traversal from the leaf nodes (ingates) to the root node (sprue), sequentially extracting parameters. This ensures that each node is properly connected and meets process requirements. To facilitate this reverse traversal, each node must store a reference to its parent node for backtracking. On the other hand, to clearly represent the flow direction of molten metal during casting, the DAG storage structure needs to allow easy traversal from the root to each child node. Therefore, each node must also store references to its child nodes.
- Separating edge attributes from node attributes: While edge attributes (e.g., information about connections via filters or risers) are necessary, they are accessed relatively infrequently. By separating edge attributes from node attributes, unnecessary redundant storage and queries can be minimized, improving system flexibility. Additionally, during data modification, nodes and edges can be updated and extended independently based on specific needs.

**Figure 8:**
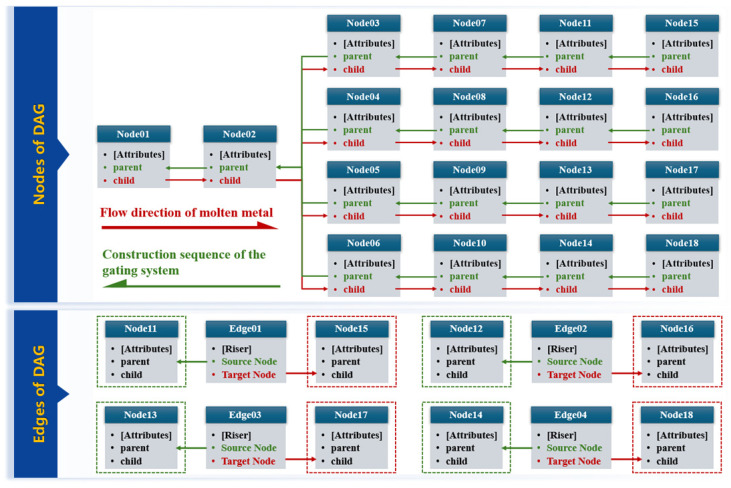
DAG data-storage structure of the axle housing’s gating system.

The DAG data-storage structure of the gating system for the axle housing part is shown in Figure 8. This gating system consists of eighteen nodes (pipe segments) and four specially connected edges (risers). The information in the DAG can be divided into two main parts: the node set and the edge set. In the node set, each node primarily stores three core elements: the attributes of the node, references to its parent node, and references to its child nodes. Through these reference relationships, the nodes are sequentially connected to form the complete structure of the graph. The red arrows between the nodes represent the flow direction of molten metal, which flows from the root node along the red arrows to each terminal child node. This visually demonstrates the flow path of molten metal entering the gating system from the sprue, flowing through each pipe segment, and ultimately reaching the mold cavity via the ingates. The green arrows represent the parsing sequence when constructing the gating system model for a new part based on the DAG data of a reference source part. Following the direction of the green arrows, node information is extracted and parsed sequentially. Based on these data, the 3D models of the various pipe segments in the gating system are gradually constructed on the new part. In the edge set, each edge represents a special connection between pipe segments, primarily riser connections in this case. The edge stores attribute information about the riser, such as its type, geometric parameters, etc. It also records the node IDs of the two pipe segments connected by the riser, clearly defining their upstream and downstream relationships.

**Figure 9:**
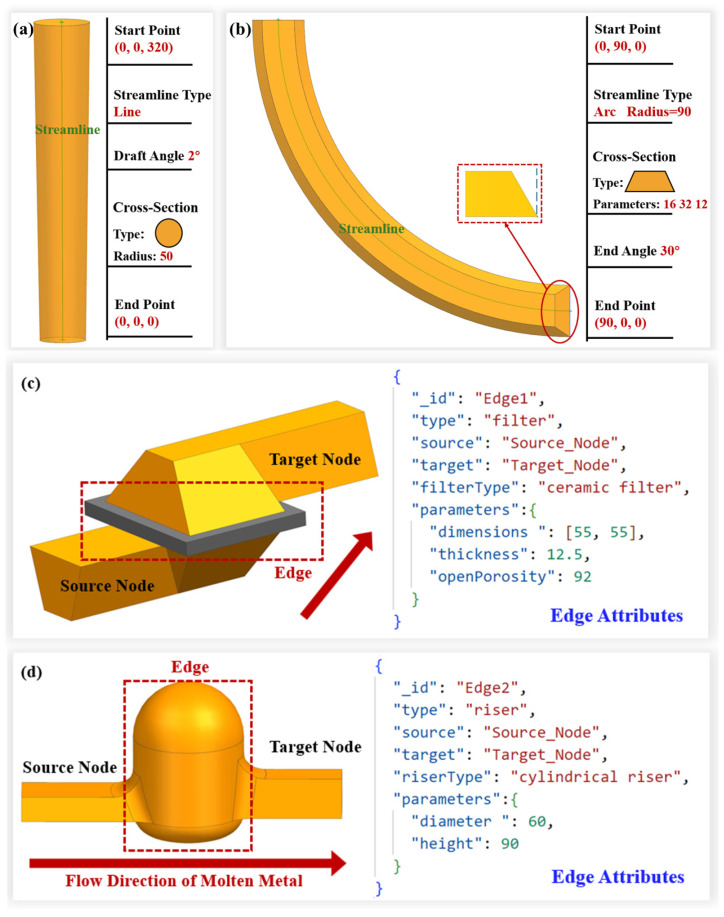
Attributes of nodes and edges: (**a**) node attributes corresponding to straight pipeline segments; (**b**) node attributes corresponding to curved pipeline segments; (**c**) filter-type edge attributes; (**d**) riser-type edge attributes.

In the enhanced adjacency list model, each node’s attributes encompass fundamental information, including cross-sectional dimensions, streamline type, and the coordinates of the start and end points. For specific pipe segments within the gating system, node attributes may be augmented to include supplementary data, such as draft angles and end-face tilt angles, to meet diverse process requirements. The typical attribute information stored in each node is illustrated in Figure 9a,b.

The types of edges primarily include connections such as filters, risers, and slag traps. For filter-type edges, the attributes must store data including the filter’s dimensions, thickness, material, and porosity, as shown in Figure 9c. For riser-type edges, the edge attributes include the riser type and specific dimensional values, as depicted in Figure 9d.

The objective of parametric modeling for the gating system is to retain parameters that represent the essential attributes of the system, providing a basis for intelligent and automated process design. A significant application scenario is the rapid construction of a new part’s gating system based on the DAG data model of a similar source part. The data within the DAG must not only intuitively reflect the conventional 3D modeling process of the gating system but also be tightly associated with the gating system’s transfer algorithms to facilitate automated functions such as position calculation, model construction, and data optimization by the computer. Consequently, when establishing the attributes of nodes or edges, it is essential to fully consider the requirements of the transfer process and supplement the previously described fundamental properties as necessary.

**Figure 10:**
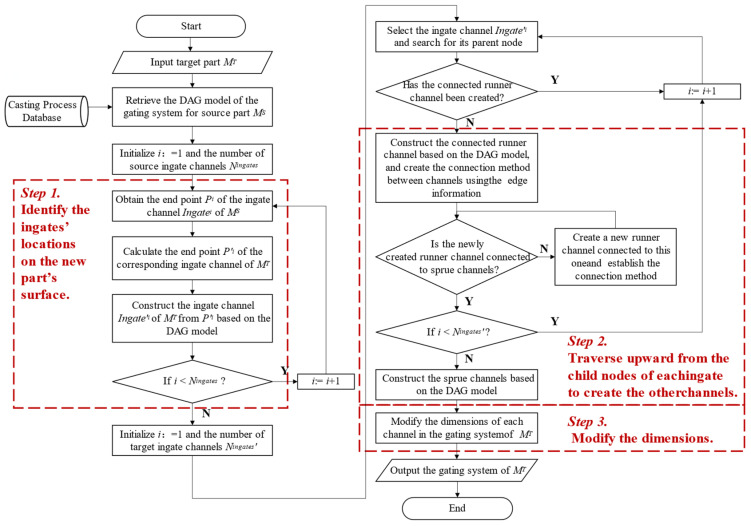
The transfer method for gating system designing between similar parts.

The prerequisite for transfer is that the source and target parts are sufficiently similar, ensuring that the corresponding gating system DAG structures are also largely consistent. The procedure for creating a new part’s gating system based on the DAG structure of a source part is illustrated in Figure 10. First, under the premise of ensuring process similarity, the coordinates of the ingate outlets on the surface of the new part are calculated based on the positional information of the ingate outlets from the source part (i.e., the endpoints of the streamline in the ingate pipeline segment). Subsequently, utilizing these coordinates along with the data from the source part’s gating system DAG model, all ingates for the new part are constructed. Then, starting from each ingate pipe of the new part, the nodes in the DAG model are traversed upward, and the corresponding pipeline segments and connections are constructed using the attribute information of the nodes and edges, ultimately forming the entire gating system. Ultimately, the cross-sectional dimensions of all pipelines are modified according to the algorithm presented in [24].

From this transfer process, it is evident that the critical step in the gating system design is to determine the placement of the gating system on the new part (i.e., the position of the ingate outlets). Without this, it would be impossible to reliably construct the ingate and other pipes. Therefore, to ensure the feasibility of the transfer process, the coordinates and geometric features of the ingate endpoints must be stored in the DAG model of the source part’s gating system. This information provides a reference for determining and establishing the ingate location on the new part. Algorithm 1 details the procedures for calculating the location of the corresponding ingate on the new target part’s surface. First, the CPCA algorithm proposed by Vranic [25] is applied to the models (in STL format) of both the source and target parts for pose normalization, aligning them in a unified reference coordinate system. Then, based on a given threshold T, a cluster of triangular facets $C_{\mathrm{candidate}}$ is identified on the surface of the new target part, in proximity to the endpoint coordinate P of an ingate streamline within the source part’s gating system. Meanwhile, the closest point $P_{i}^{\prime }$ on each triangular facet to P is calculated. Finally, the optimal $P_{i}^{\prime }$ is selected as the ideal location for the new part’s corresponding ingate, based on the geometric similarity between point P and each $P_{i}^{\prime }$. Equation (6) is used to measure the local geometric similarity between two points, where a smaller error ${\mathrm{Err}}_{i}$ indicate greater similarity.

(6) $${\mathrm{Err}}_{i}=\omega_{1}\cdot \|N_{i}{\;-\;N}_{\mathrm{source}}\|+\omega_{2}\cdot \left|\sqrt{K_{i}^{2}+H_{i}^{2}}-\sqrt{K_{\mathrm{source}}^{2}+H_{\mathrm{source}}^{2}}\right|$$

where $N_{\mathrm{source}}$, $K_{\mathrm{source}}$, $H_{\mathrm{source}}$ represent the normal vector, Gaussian curvature, and mean curvature at point P, respectively, and $N_{i},{\;K}_{i},{\;H}_{i}$ represent the normal vector, Gaussian curvature, and mean curvature at point $P_{i}^{\prime }$, respectively.

According to the aforementioned information, specific key attribute information must be stored in the terminal nodes of the DAG (i.e., the nodes representing the ingate pipe) in order for the computer to automatically determine the best location for the new part’s ingate during the gating system transfer. These data encompass the coordinates of the endpoint, normal vector, Gaussian curvature, and mean curvature. These properties are essential for computing the geometric similarity among several points, facilitating the accurate identification of the corresponding ingate’s location on the new part.
**Algorithm 1** $\mathrm{Find}\;\mathrm{point}\;P_{optimal}^{\prime }$ on the target part to serve as the end of an ingate.**Input:** STL_model: List of triangular faces $\left\{Face_{i}:\left(PointA_{i},\;PointB_{i},PointC_{i}\right)\right\}$ P: The endpoint of the streamline in an ingate of the source part’s process T: The threshold of distance $N_{source}$: The normal vector at point P. $H_{source}$: The mean curvature at point P. $K_{source}$: The Gaussian curvature at point P.1:Initialize List Centers=[]
2:Initialize Dictionary Face_Map={}
3:**For** $Face_{i}$ in STL_model **do**4: Calculate the center point $C_{i}$ of $Face_{i}$
5: $Centers.append\left(C_{i}\right)$
6: $Face\_Map\left[C_{i}\right]=Face_{i}$
7:**End For**8:Build K-D Tree kdTree using Centers
9:The cluster of center points closest to P
$C_{candidate}=kdTree.rangeQuery\left(P,T\right)$
10:Initialize List Candidate_Point_Info=[]
11:**For** $C_{i}$ in $C_{candidate}$ **do**12: $Face_{i}=Face\_Map\left[C_{i}\right]$
13:Calculate the projection point $P_{i}^{\prime }$ of P on the $Face_{i}$ plane14:**If** $P_{i}^{\prime }$ is outside of $Face_{i}$ **then**15:Choose the vertex of $Face_{i}$ that is closet to P as the new $P_{i}^{\prime }$
16:**End If**17: $Candidate\_Point\_Info.append\left(P_{i}^{\prime },Face_{i}\right)$
18:**End For**19:Initialize min_total_error=∞
20:Initialize $P_{optimal}^{\prime }=None$
21:**For** $\left(P_{i}^{\prime },Face_{i}\right)$ in $Candidate\_P^{\prime }$ **do**22:Approximate the normal vector $N_{i}$ of $P_{i}^{\prime }$ by the normal vector of $Face_{i}$
23:Compute the mean curvature $H_{i}$ and Gaussian curvature $K_{i}$ at $P^{\prime }$ using barycentric coordinate interpolation24:Substitute $N_{source}$, $H_{source}$, $K_{source}$, $N_{i}$, $H_{i}$, $K_{i}$ to calculate the total error $Err_{i}$ using Equation (1)25:**If** $\left(Err_{i}<min\_total\_error\right)$ **then**26: $min\_total\_error=Err_{i}$
27: $P_{optimal}^{\prime }=P_{i}^{\prime }$
28:**End If**29:**End For****Output:** The position of the optimal point $P_{optimal}^{\prime }$ on the STL surface of the target part.

## 4. Visual Verification of Casting Process Database Functionality

### 4.1. Rapid Process Design for the New Part

Taking the procedure of designing a new axle housing part’s casting process based on CBR as an example, the application of the database is illustrated. The new part is a ductile iron axle housing, weighing 57.72 kg, with dimensions of 1280 mm × 383 mm × 270 mm.

**Figure 11:**
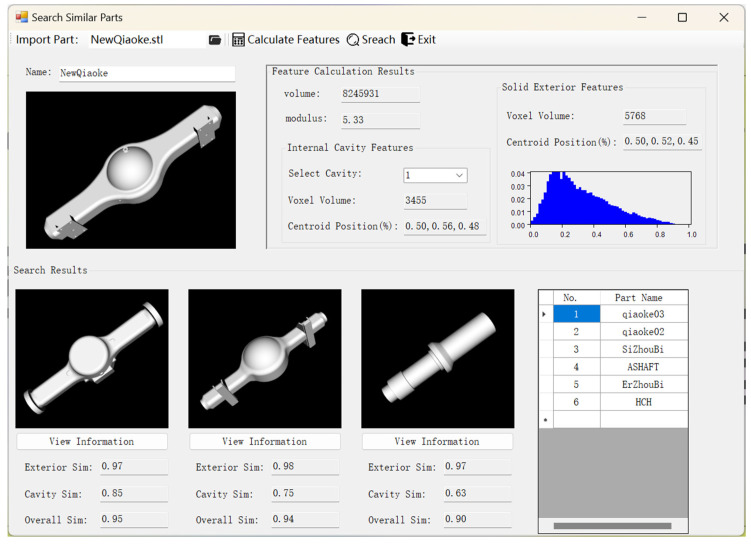
The interface for retrieving similar parts.

First, several parts similar to the new part were retrieved from the database. This process involved extracting the structural shape features of the new part and calculating the similarity between these features and those of the stored source parts in the database, followed by ranking the results. Figure 11 shows the interface for retrieving similar parts, which displays the feature calculation results of the new part, including volume, modulus, solid exterior features, and internal cavity features. The D2 distances are presented in the form of a frequency distribution histogram. The search results visually show the three most similar parts, facilitating manual comparison and final selection.

**Figure 12:**
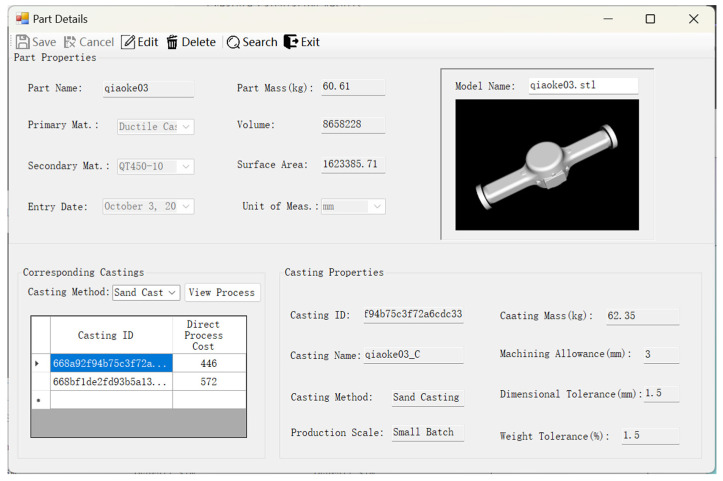
The primary information of the reference source part.

The part exhibiting the greatest similarity, displayed as the initial result in the search, was designated as the reference part for the process design of the new part. Figure 12 presents the primary information of the reference source part, including material type, volume, mass, and other relevant data. At the bottom of the interface, all castings associated with different casting methods for this part in the database are enumerated, along with the direct cost of the corresponding process. The basic properties of a particular casting are presented on the right.

**Figure 13:**
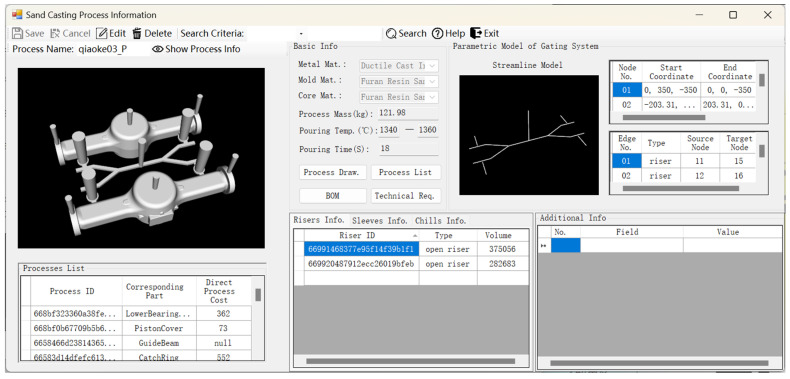
Process information display interface.

The optimum process for the reference source part was selected based on the direct process cost indicator, and the process information is displayed in Figure 13. The interface presents process data organized into four primary modules: basic information, the parametric model of the gating system, standard component information, and additional information. The basic information module includes frequently utilized process design data for sand casting, together with process drawings, process cards, bills of materials, and technical specifications. The parametric model of the gating system primarily represents the attribute information of the nodes and edges within the graph structure that defines the gating system. This includes the start and end point coordinates of each node, the cross-sectional dimensions of each node, the types of edges, and their relationships with other nodes. Additionally, the streamline model of the gating system is visually displayed in the module. The standard component information comprises the materials and dimensions of frequently utilized components, such as risers, riser sleeves, chills, etc. Additional information may consist of any custom details that enhance the process description.

**Figure 14:**
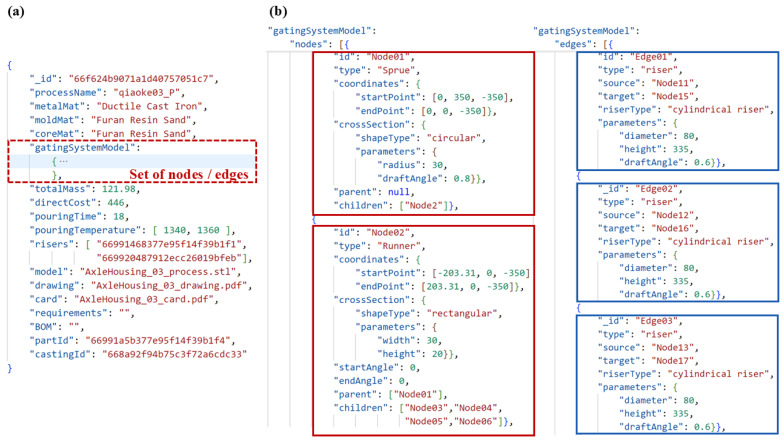
(**a**) The storage contents of the process data unit; (**b**) the specific parametric model information of the gating system. Red boxes represent nodes, and blue boxes represent edges, respectively.

In the MongoDB database, the data unit corresponding to this process stores information as shown in Figure 14a, including key data such as material information, pouring information, and the storage names of process-related files. The parametric model information of the gating system is shown in Figure 14b, which consists of a set of nodes and a set of edges. Each red box represents a node, storing the dimension, positional parameters of the corresponding pipe segment, and the IDs of the two connected pipe segments. Each blue box represents an edge, storing connection details, including specific information about the joints of the connected pipe segments. For this process, risers are set at four pipe connections, and the attributes of the edges record detailed riser information, such as diameter and height. Additionally, by associating the two nodes connected by an edge, the specific location of the risers can be indirectly determined.

**Figure 15:**
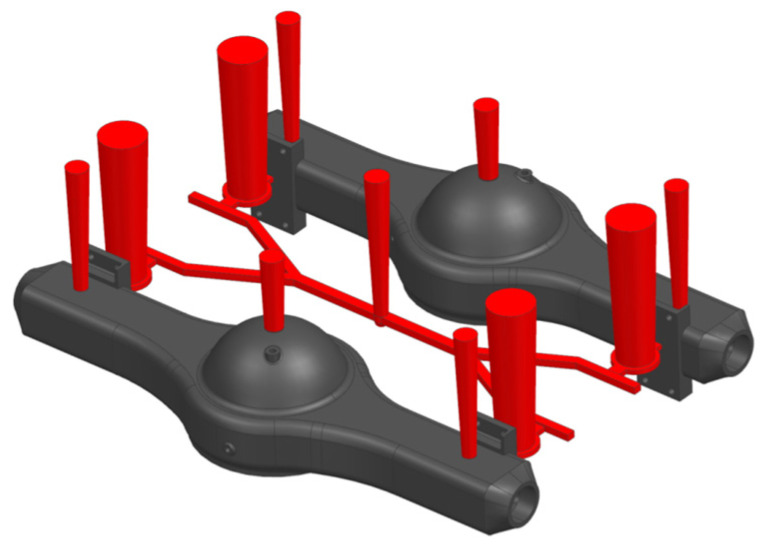
The process of the new part.

After extracting the parametric model of the gating system from the database, the cross-sectional dimensions of each pipe need to be recalculated using the migration algorithm described in the literature [24]. This involves updating the relevant parameters in the “parameter” field of the node data unit in Figure 14b. Once the adjustments are completed, the modified parametric model of the gating system will be used as the gating system model for the new part, enabling the rapid generation of a 3D solid model of the gating system for the new part. The final process created for the new part is shown in Figure 15.

### 4.2. Simulation Results and Analysis

**Figure 16:**
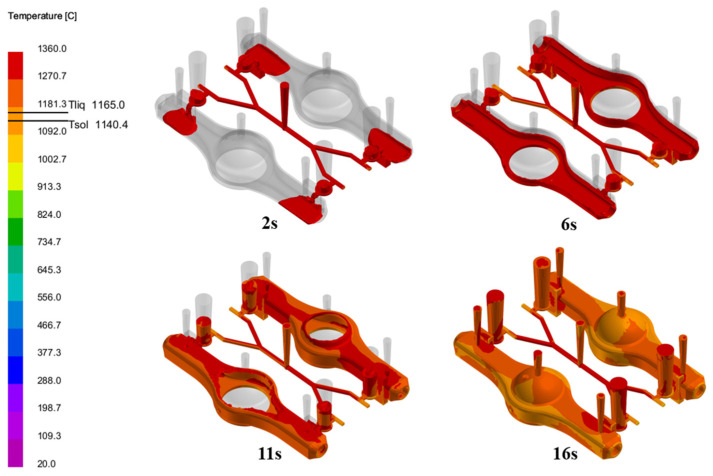
Simulation results of the new part’s casting process.

**Figure 17:**
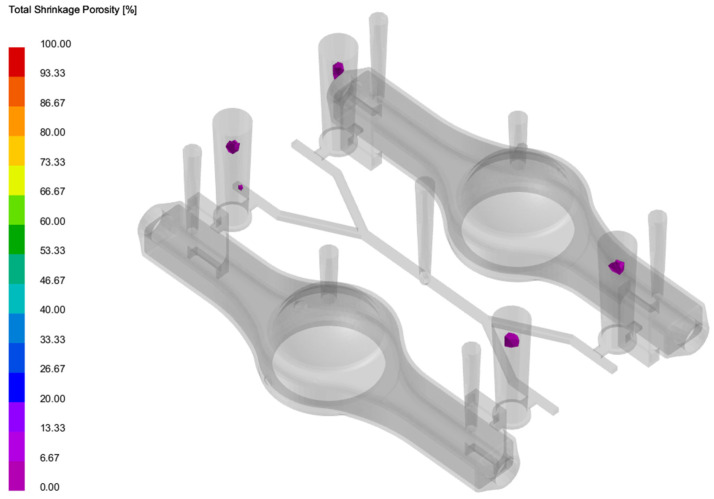
Shrinkage porosity in the process.

The process of the new part was simulated, with the results of molten metal filling shown in Figure 16. It can be observed that the flow of molten metal was relatively smooth, with no significant turbulence that could cause air entrapment or slag inclusion, and the mold cavity was ultimately filled completely. The solidification simulation results are shown in Figure 17, where the purple areas represent regions prone to shrinkage porosity and cavities, which are critical solidification defects. The results indicate that these defects are all concentrated within the risers, with no defects present inside the casting itself. Therefore, the simulation results comprehensively demonstrate that using a database combined with a CBR approach for the rapid design of a new part’s process is both feasible and effective.

The traditional casting process design workflow is highly dependent on designers’ experience and is time-consuming when retrieving data and performing calculations. Enterprise research reveals that in traditional workflows, designers must sequentially determine the placement of the gating system, the length and cross-sectional dimensions of each pipe, as well as the size and placement of risers. Simultaneously, the quality of the design plan cannot typically be fully guaranteed in the early stages and requires feedback and adjustments based on casting simulations or actual production results, further extending the design cycle. For small- to medium-sized parts, such as axle housings, as an example, the process design—including feedback and adjustments—typically requires approximately 4 h. For less experienced designers, the time required is notably longer.

In contrast, the visualization application results of the casting process database in this study demonstrate that the CBR-based design framework, combined with database technology, significantly reduces process design time by offering functions such as the rapid retrieval of similar parts, cost-based optimal process recommendations, convenient process information queries, and parametric gating system-model management. The design time for new parts can be reduced to less than 20 min, and with further integration into CAD technologies, the design time is expected to decrease even further. Moreover, the design results were validated through simulation, proving the effectiveness and reliability of this method. Compared to traditional methods, this framework reduces design time by more than 90%, significantly improving design efficiency while maintaining accuracy. Overall, it provides an efficient solution for casting process design.

## 5. Conclusions and Future Work

### 5.1. Conclusions

Casting process design often encounters challenges related to efficiency, accuracy, and stability. To address these issues, this study has established a casting process database built on the parameterization of mature process models. The database framework was designed to associate diverse types of information involved in process design, enabling the comprehensive storage and detailed description of complete process cases. Parametric features of parts and process models were analyzed and extracted; the structural parameters of parts were calculated and stored based on analyses of part geometries; a direct cost associated with the process was recorded, enabling the ranking and comparison of various process plans for the same part; and a parametric model of the gating system was stored to facilitate the transfer of mature processes to new parts. By implementing parametric representation, the need for manual definitions is significantly reduced, improving the digitalization and management of process information while providing essential data support for the rapid design of new processes through the reuse of proven designs. Finally, the functionality of the casting process database was tested by designing the process of a new part using CBR as an example. The results indicate that the database’s functionality significantly improves the efficiency and accuracy of process design, optimizing the reuse of design knowledge and experience. Consequently, this database plays a crucial role in advancing the design of intelligent and information-driven casting processes.

### 5.2. Future Work

This study focuses on the construction and explanation of an overall framework for rapid process design based on CBR combined with database technology, and it preliminarily validates the completeness and feasibility of the system workflow. However, test results from our previous study indicate that the part-retrieval method used in this database demonstrates high applicability for parts with relatively simple shapes, such as cylindrical or disk-shaped parts, but exhibits certain limitations when applied to parts with complex geometries. Therefore, future research will prioritize optimizing the description methods for geometric shapes and the structural features of parts, for example, introducing more distinctive and adaptive geometric descriptors to enhance the resolution of model feature representation and improve retrieval accuracy. Additionally, the applicability of the database system in various manufacturing scenarios will be explored in greater depth. This includes optimizing process feature extraction and matching algorithms tailored to different manufacturing conditions, further enhancing the system’s generalizability and intelligence. By doing so, it aims to better meet the demands of complex process design.

The ultimate goal of the study is to develop an intelligent casting process modeling system based on database and CAD technologies. This system aims to achieve automated casting process modeling—including gating systems, risers, riser sleeves, and chill placement—by exporting specifically formatted data from a parametric database and importing it into a CAD system. A comprehensive design framework has already been proposed, addressing the structural construction of the database, the design of core storage content, and the visualization of key functions. However, the full implementation of the system still faces numerous challenges, such as further optimization of stored design content and the intelligent design of risers and chills to accommodate subtle differences in part structures. Future work will focus on the efficient integration of the database with the CAD system. In our current research, through the output of parametric data formats from the process database, the preliminary implementation of automated modeling has been achieved in NX 3D modeling software (Version 1980, Siemens NX). For instance, riser information units exported from the standard component module of the database can drive automatic riser modeling through parameters. Similarly, a gating system’s parametric model, stored in an XML format with a tree structure, can also be automatically constructed through programmatic parsing and feature modeling techniques. These attempts have validated the feasibility of integrating the database with the CAD system. However, achieving a systematic and complete modeling solution requires further in-depth research, enhanced software development, and functional optimization. These efforts are crucial to comprehensively improving the system’s intelligence and applicability.

## Data Availability

The original contributions presented in the study are included in the article, further inquiries can be directed to the corresponding author.
